# Bayesian network analysis of antidepressant treatment trajectories

**DOI:** 10.1038/s41598-023-35508-7

**Published:** 2023-05-24

**Authors:** Rosanne J. Turner, Karin Hagoort, Rosa J. Meijer, Femke Coenen, Floortje E. Scheepers

**Affiliations:** 1 Department of Psychiatry, UMC Utrecht Brain Center, University Medical Center Utrecht, Utrecht University, 3584 CX Utrecht, The Netherlands; 2 Machine Learning Group, CWI (National Research Institute for Mathematics and Computer Science), Amsterdam, The Netherlands; 3 Data Science Department, Parnassia Groep, The Hague, The Netherlands

**Keywords:** Psychiatric disorders, Depression

## Abstract

It is currently difficult to successfully choose the correct type of antidepressant for individual patients. To discover patterns in patient characteristics, treatment choices and outcomes, we performed retrospective Bayesian network analysis combined with natural language processing (NLP). This study was conducted at two mental healthcare facilities in the Netherlands. Adult patients admitted and treated with antidepressants between 2014 and 2020 were included. Outcome measures were antidepressant continuation, prescription duration and four treatment outcome topics: core complaints, social functioning, general well-being and patient experience, extracted through NLP of clinical notes. Combined with patient and treatment characteristics, Bayesian networks were constructed at both facilities and compared. Antidepressant choices were continued in 66% and 89% of antidepressant trajectories. Score-based network analysis revealed 28 dependencies between treatment choices, patient characteristics and outcomes. Treatment outcomes and prescription duration were tightly intertwined and interacted with antipsychotics and benzodiazepine co-medication. Tricyclic antidepressant prescription and depressive disorder were important predictors for antidepressant continuation. We show a feasible way of pattern discovery in psychiatry data, through combining network analysis with NLP. Further research should explore the found patterns in patient characteristics, treatment choices and outcomes prospectively, and the possibility of translating these into a tool for clinical decision support.

## Introduction

Patients seeking treatment for severe depression symptoms often have a long trajectory ahead of them; only approximately one third continues their medication of first choice^1,2^ and about 30 percent has still not achieved remission after four treatment steps^3^. Meanwhile, the contribution of mental health disorders to the global burden of disease is substantial^4^. Despite the limitations, pharmacological treatment of severe depression is still the most common treatment choice. Since it is still difficult to predict the response to a specific antidepressant type in an individual, the prescription process is one of trial and error. For a patient this can result in unnecessary and possibly harmful side effects and delayed recovery. Especially challenging in the prescription of antidepressants is that both the choice of the antidepressant and the response are influenced by multiple variables relating to the prescriber, the patient, illness characteristics and the drug itself^5^. Insights into the interactions between these factors and their effects on treatment outcomes could allow greater precision in the choice of an antidepressant for a given patient, but are currently lacking^6^.

To empower patients and clinician during treatment choices, the multi-faceted, non-binary aspects of psychiatric care are hard, but essential to account for^7^. During the last decade many machine learning models with the aim of personalizing treatment recommendations for patients with symptoms of depression have been developed^8^. However, little has changed in actual clinical psychiatry practice yet, perhaps because of the “black box” nature of most clinical machine learning models^9^.

Network analysis is a promising candidate from the joint field of statistical learning and machine learning that could potentially offer the desired multi-faceted insights into psychopathology in an explainable and transparent manner^10^. It comprises of methods of data analysis where dependencies and/or causal pathways between all variables in a dataset are learned and visualized^11^. Because mental health syndromes often present as a collection of tightly intertwined signs and symptoms, which sustain and influence each other and can be intervened on through multiple pathways, network analysis appears especially apt for capturing these concepts.

Previous studies on network analysis in mental health mainly focused on modelling symptom networks and yielded promising results. A study with a penalized Gaussian graphical model, including 1029 participants and 16 depression and anxiety symptoms, resulted in stable networks^12^. In a greedy search Bayesian network approach with a relatively small sample size of 353 subjects where relations between 10 stress-related variables were investigated, moderate classification accuracy of the network was achieved^13^. Network analyses studies with similar sample sizes and numbers of variables on obsessive–compulsive disorder and depression, and suicidal ideation (408 and 336) also revealed key gateway symptoms influencing symptom clusters^14,15^. A pilot with personalized feedback to patients through symptom network analysis showed promising results with respect to increasing a patient’s understanding of their psychopathology^16^.

The above-mentioned studies illustrate the aptness of network analysis for showing and interpreting associations between symptoms. In the future, a tool for explainable personalized insights into antidepressant recommendations based on these kinds of networks could potentially be of significant value in clinical decision making. To work toward this goal, for this study, we intended to explore if treatment characteristics (antidepressant choices and co-medication), patient characteristics and treatment outcomes in addition to symptom scores can be incorporated in network analysis, using retrospective data from two mental healthcare facilities in the Netherlands. To extract information on mental health symptoms and treatment outcomes from the retrospective data, the network analysis was combined with a natural language processing (NLP) model^17^.

Since our end goal is to develop a tool for explainable personalized insights into antidepressant recommendations we were primarily interested in causal paths and discovering (conditional) dependence relations among patient characteristics, treatment choices and outcomes. Hence, we have chosen to perform a Bayesian network (BN) analysis instead of a partial correlation network analysis or Markov random field analysis^18^. The final BN, the found dependencies and predictions for hypothetical patients were compared to expert opinion to assess the potential of the model for future implementation in a tool for clinical decision support.

## Methods

### Main units of analysis

Main units of analysis were first-time inpatient antidepressant treatment trajectories at participating mental health care facilities; consecutive prescriptions for one type of antidepressant were viewed as a single treatment trajectory. New prescriptions for the same type of antidepressant that started within 30 days after the old prescription were viewed as belonging to the same trajectory.

Antidepressant treatment trajectories between 2014 and 2020 at two mental healthcare facilities involved were included. The first mental healthcare facility, Parnassia Group (PG), provides basic and specialized services for prevention, treatment (inpatient and outpatient), support and care after treatment. The second facility, UMC Utrecht (UMCU), is an academic specialized facility for tertiary care. As PG and UMCU deliver care in different regions in the Netherlands, the probability of overlap in patient populations is negligible.

To ensure a homogeneous patient population, only trajectories with (partial) inpatient treatment were included. We ultimately aim to assist a broader group of patients than only those with a clear-cut classification fitting the Diagnostic and Statistical Manual of Mental Disorders (DSM) categories. Therefore, all antidepressant trajectories were included regardless of DSM classification^19^. However, to keep the populations from both facilities comparable we did not include patients with addiction as a primary diagnosis, since PG includes a few clinics specialized in addiction treatment and UMCU does not, and addiction as a primary diagnosis has a dominant impact on all interventions^20^. Patients with addiction as a secondary diagnosis were included, to still enable investigating the possible interactions between depressive symptoms, choice of antidepressant and treatment with disulfiram.

### Predictor variables

Predictor variables available at the start of (or becoming available during) the treatment trajectories comprised of gender, age, antidepressant type, co-medication, psychiatric (co-)morbidities according to the DSM classification system and global assessment of functioning (GAF) scores as registered in the DSM classification system. Antidepressant types were grouped into selective serotonin reuptake inhibitors (SSRI), non-selective serotonin reuptake inhibitors (nSSRI), tricyclic antidepressants (TriCA), tetracyclic antidepressants such as mirtazapine and mianserine (TetraCA), monoamine oxidase inhibitors (MAOI) and a remainder category (other), including for example bupropion (for a full overview, see Supplementary Table 1). Co-medication subgroups included in analysis were lithium, antipsychotics, tranquilizers (benzodiazepines) and disulfiram. Disulfiram was included because of its strong interactions with TriCAs^21^. As information on other forms of treatment running concurrently, such as psychotherapy, was not available in a homogeneous format within and across treatment facilities, we did not incorporate these other treatments as predictor variables. (Co-)morbidities included were depression, anxiety disorder, personality disorder and problems in the social environment. Information on all variables except GAF scores was complete; missing data on GAF scores were imputed using the MICE software in R^22^.

### Outcome variables

Acceptability and efficacy are the main categories of outcome variables in antidepressant research. In this study acceptability is operationalized in prescription duration (≥ 5 weeks indicating an “effective” duration, i.e., long enough for a treatment effect to be observed), and continuation of the antidepressant type (the final type prescribed at the mental healthcare facility during consecutive treatment for that patient). Efficacy was measured in terms of change scores on four mental health recovery themes: psychiatric core complaints, general well-being, social functioning and patient’s experience. These last four scores were extracted from doctors’ and nurses’ notes with an NLP model, as described in previous work^17^. We explicitly chose not to use Hamilton scores as outcomes in this study, as those only focus on symptom reduction and are not systematically registered during routine clinical care in the Netherlands. Concisely, all available clinical notes during the patients’ antidepressant treatment trajectories were screened for sentences concerning moments of change on one of the four themes, mentioned in a correct context (including, for example, “Today, the patient’s mood significantly improved” but not “last year, after their grandmother died, the patient’s mood declined”). The detected words were then combined with a sentiment score (1 or − 1 for each detected word), a positive score indicating a positive change and vice versa, and a mean score for the entire treatment trajectory was calculated for each theme.

### Medication doses

At least 24 different antidepressants were prescribed at PG and UMC Utrecht between 2014 and 2020. To ensure faulty entries in the electronic patient files were not included in the dataset, prescriptions where less than half of the minimal therapeutic dose according to the Dutch national standards of care was prescribed were excluded^23^. These minimal doses as listed May 2020 are summarized in Supplementary Table 2. Further, prescriptions exceeding five times the maximal therapeutic dose were excluded as well, as these can only be faulty entries in the electronic patient records.

### Bayesian network analysis

All analyses were performed with R (version 4.0.0) using the “bnlearn” package^24^, and network visualizations were constructed with the “qgraph” package^25^.

**Figure 1:**
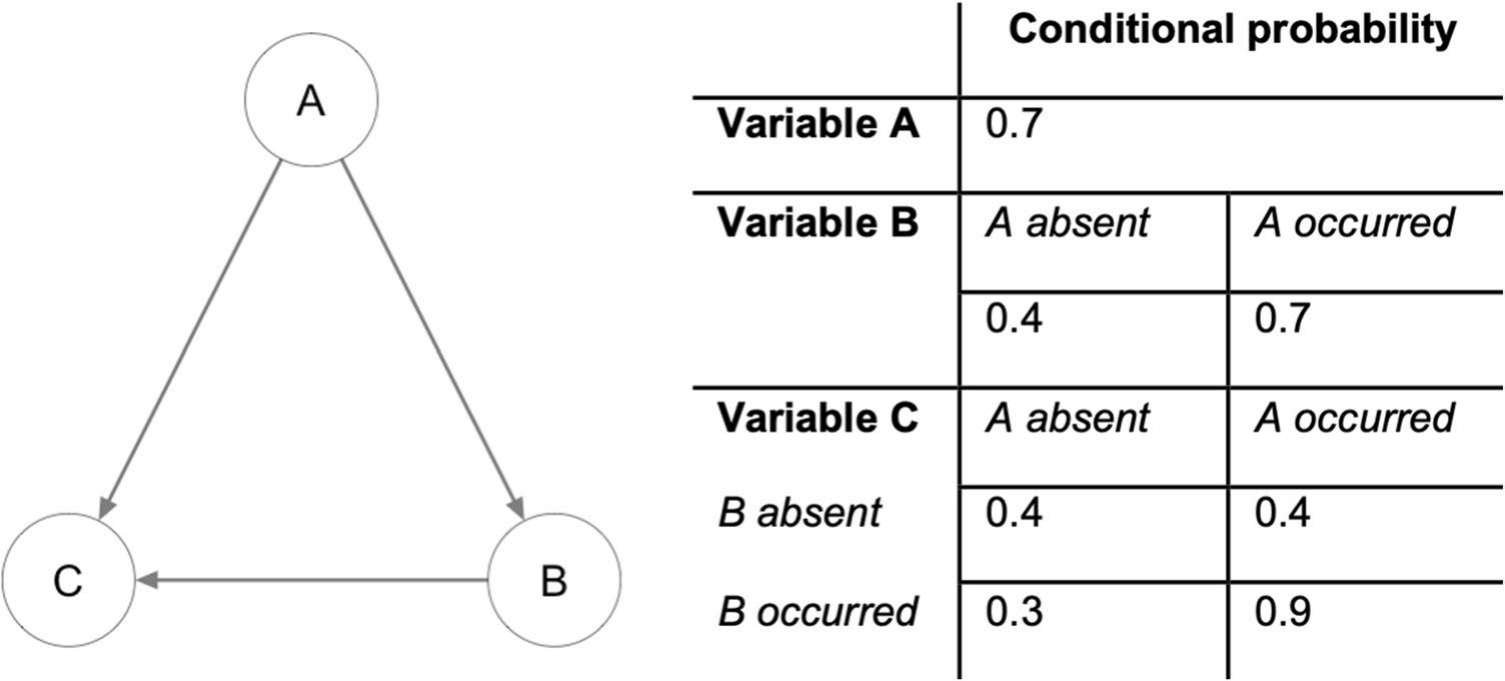
Toy example of a depiction of the structure of a Bayesian network with three binary variables and the corresponding predictive model: in this case, a conditional probability table.

A BN is a representation of the presence and strength of dependencies between all variables in a dataset (these could be predictive and/or causal, see further below). A BN represents qualitative and quantitative information. It includes the structure of the dependencies (sometimes also called relations or associations), often depicted in a schematic figure called a graph, and the corresponding quantitative model built with these dependencies. For example, in the toy BN depicted in Fig. 1, the dependencies between the three variables are indicated with arrows in the graph on the left, and the corresponding model quantification in the form of a conditional probability table is depicted on the right. Learning a BN from data and/ or expert knowledge also follows these two stages: first one performs structure learning, identifying the relevant dependencies and their direction; and secondly parameter learning, estimating the parameters that quantify the dependencies^18,24^.

Learning the structure of the BN teaches us which other variables in the dataset influence the probability that a variable takes on a certain value. Going back to the toy example, variable B has one incoming arrow, from variable A, indicating that we can write our prediction of the probability that B will occur in terms of A, “B depends on A”. Variable C has two incoming arrows, indicating that “C depends on both A and B”. Conveniently, this dependency also gives us information in the other direction^11^: if we have observed that B has taken on the value 1, we have a better estimate of which value A has compared to the scenario where we do not have any information at all, which we will also use for making predictions in the final part of this paper.

In general, there are two approaches for structure learning: constraint-based and score-based. Constraint-based learning aims to optimize the learning process to discover conditional dependencies between variables based on statistical inference and hypothesis testing. Score-based structure learning is aimed at optimizing the predictions the model makes for the data (formally: the likelihood of the joint probability distribution of all variables in the dataset). In this paper, both approaches were applied (“pcStable” was used as a constraint-based method, and “tabu” as a score-based method). To reduce the possibility of including spurious dependencies, model averaging was performed with bootstrap resampling, with 100 iterations. To ensure stability of the found associations, only edges that appeared in more than 85% of bootstrap samples were included in the averaged network^18^. To investigate stability of associations across the two mental healthcare facilities, bootstrapped averaged networks were obtained at both facilities with both methods and compared with respect to found dependencies between variables.

To quantify the model, the current version of the Bayesian network software we used only offers the possibility of incorporating discrete variables as predictors of discrete variables; discrete variables cannot depend on continuous variables, potentially limiting the structures that can be found. To account for this, all variables were converted to binary variables. Age and GAF scores were compared to their respective median value at PG to ensure comparability across locations, with age being converted to older or younger than 48 (the median age in the PG data), and GAF being higher or lower than 50. Antidepressant prescriptions durations were converted to ≥ 5 weeks or < 5 weeks (minimum time for an expected clinical effect), and mean sentiment scores being positive (≥ 0) or negative (< 0).

Model parameters were fitted for the average model according to their maximum likelihood estimators and the resulting conditional probability tables were recorded. A toy example of such a table is included in Fig. 1. For example, because B depends on A, it can be observed that the probability of B occurring increases from 0.4 to 0.7 if we know A has occurred. C depends on both A and B, and it can also be observed that the model captures an interaction between A and B: in the absence of B, the effect of A on C disappears. Such model predictions in the presence or absence of information on specific hypothetical patient and treatment characteristics were also generated for the final network using the logic sampling functionality in the bnlearn package and compared to expert (FS) knowledge.

### Ethics statement

This study (number 22–705/DB) was assessed and approved to not fall under the scope of the Medical Research Involving Human Subjects Act (WMO) by MREC NedMec: a recognized medical research ethics committee to which the Antoni van Leeuwenhoek, the Princess Máxima Center for pediatric oncology and the UMC Utrecht are affiliated. Complying with the guidelines issued by the MREC NedMec for research not falling under the Medical Research Involving Human Subjects Act (WMO), informed consent was waived by a quality officer from the research and ethics board of the Brain Center of UMC Utrecht on behalf of the MREC NedMec. It was deemed a disproportional effort to obtain informed consent of each individual patient because of the retrospective nature of the study and number of patients, of which many could not be contacted anymore because they continued their treatment elsewhere. However, the centers where this study was carried out uses an opt-out policy for patients who do not want their data to be used for research. Only data from patients who did not object to the use of their routinely collected electronic health record data were analyzed. According to Dutch national guidelines, the board of each university medical center is responsible for research quality control^26^. For this study, the protocol was approved by a quality officer from the research and ethics board of the Brain Center of UMC Utrecht, appointed by the board of UMC Utrecht. This study conforms to the declaration of Helsinki for ethical principles involving human participants. To assure patients’ privacy data were de-identified, for which the DEDUCE software was used^27^.

## Results

4808 and 735 trajectories of patients with first-time inpatient antidepressant prescriptions were included in PG and UMCU respectively. In Table 1 summary statistics of included trajectories are depicted. At PG, there is generally a long outpatient follow-up of patients, as the facility offers a wide range of levels of care: the mean period during which follow-up treatment was given at PG after the start of a first inpatient antidepressant trajectory was 1175 days (median follow-up duration: 866 days). At UMCU however, care is very specialized and patients are referred to other care facilities after dismissal: 1 month after dismissal, for 261 trajectories where patients were released into ambulatory care there still was an (ambulatory) care path at UMCU. For 174 trajectories, patients were referred to inpatient care at another facility. This resulted in a mean (inpatient) follow up duration of only 52 days after the start of antidepressant prescriptions.

**Table 1:** Overview of patient and treatment characteristics of included treatment trajectories.

Variable	PG (n = 4808)	UMCU (n = 735)
	Mean (sd) or proportion	Mean (sd) or proportion
Follow-up from start prescription (days)	1175 (804)	52.7 (59.8)
Age	48.42 (17.97)	43.861 (17.02)
Sex: female	0.577	0.615
Prescription group: MAOI	0.013	0.061
Prescription group: other	0.047	0.020
Prescription group: SSRI	0.467	0.430
Prescription group: nSSRI	0.161	0.200
Prescription group: TCA	0.177	0.261
Prescription group: TetraCA	0.172	0.060
Benzodiazepine prescription	0.643	0.848
Lithium prescription	0.081	0.165
Antipsychotics prescription	0.423	0.574
Disulfiram prescription	0.015	0.003
DSM: Depression	0.401	0.571
DSM: Personality disorder	0.268	0.242
DSM: Anxiety disorder	0.077	0.125
DSM: Social problems	0.025	0.211
GAF score at start treatment	48.03 (9.463)	33.33 (13.56)
Medication trajectory duration (days)	162.7 (234.1)	109.4 (236.0)
Continuation of antidepressant	0.663	0.894
Mean change sentiment core complaints	− 0.152 (0.752)	− 0.206 (0.703)
Mean change sentiment social functioning	0.321 (0.698)	0.464 (0.696)
Mean change sentiment well-being	0.293 (0.618)	0.162 (0.696)
Mean change sentiment experience	− 0.094 (0.681)	− 0.160 (0.590)

### Duration and continuation

The average prescription duration of the first-time antidepressant trajectories at PG was 163 days and 109 days at UMCU. At PG, 33.7% of patients switched to a different antidepressant type during follow-up, whereas at UMCU, only 10.6% of patients switched. This could partially be explained by the shorter follow-up period at UMCU, or the fact that more patients at UMCU had a history of ineffective antidepressant use. Note that the average prescription duration at UMCU exceeds the mean follow-up time, as many patients were dismissed with a prescription to continue their antidepressant use as at home or in another clinic.

Outcome measures are summarized in detail for each type of initially prescribed antidepressant in Supplementary Table 3. At both facilities, patients were most likely to continue their prescription when they started with SSRI or TriCA. Patients were most likely to switch when they started with an “other” type of antidepressant (often bupropion), a MAO inhibitor (UMCU) or a tetracyclic antidepressant (PG). Prescription durations were also the shortest for tetracyclic antidepressants, and relatively long for MAO inhibitors and nSSRIs.

**Figure 2:**
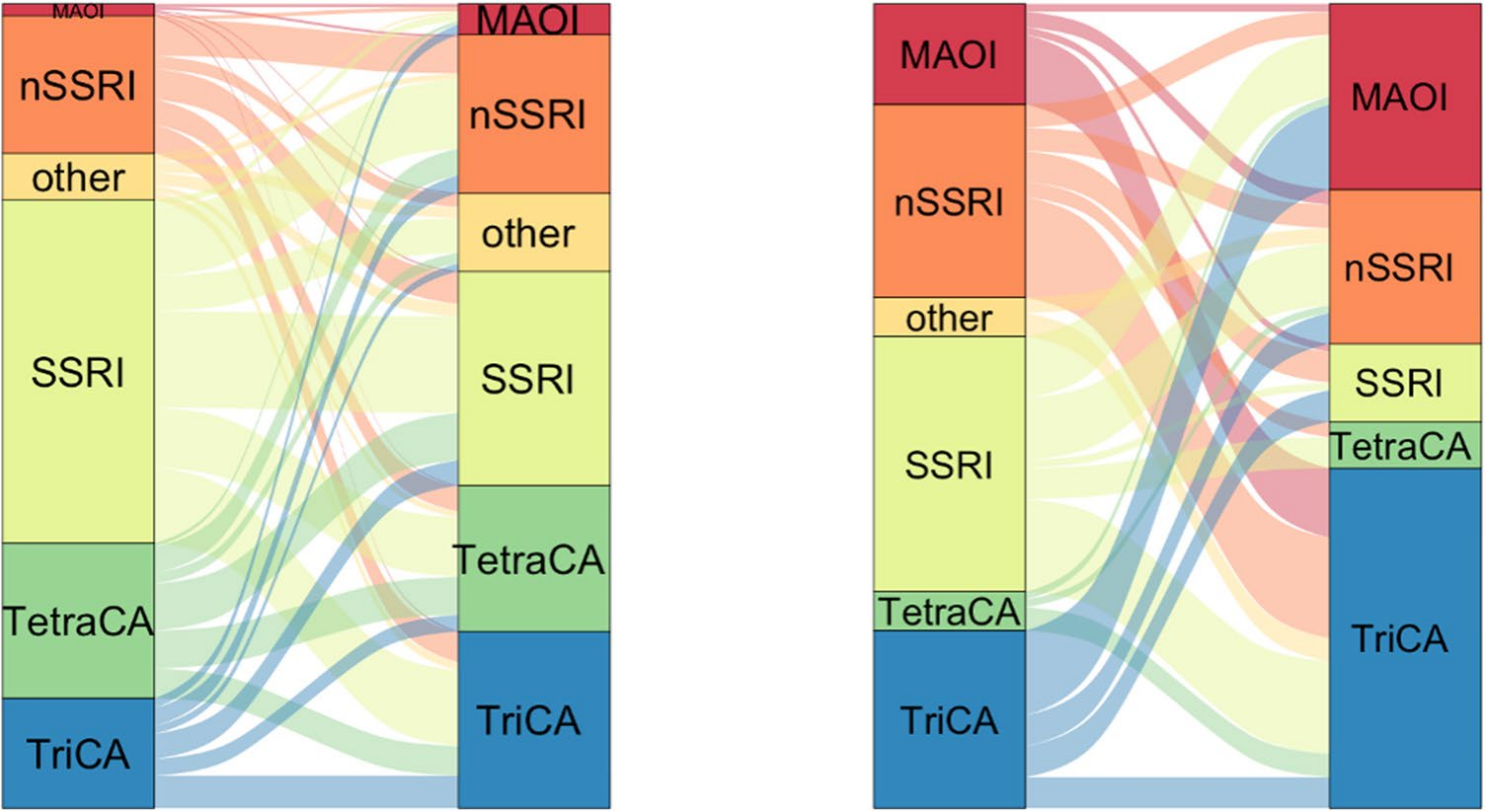
Flow diagram of antidepressant type switches for patients who did not continue their initial prescription for PG (left) and UMCU (right). Note the existence of flows from an antidepressant type to that same type during follow-up: these occur when a patient started with a single type of antidepressant and later switched to a combination of types or switched and thereafter returned to the original type. Pauses in prescriptions of the same antidepressant type were not regarded as switches.

In Fig. 2 (constructed using the ggalluvial package^28^), antidepressant type prescription switches for patients who did not continue their initial type(s) of antidepressant are depicted. At PG, SSRI is the biggest group that patients switch to, and at UMCU, patients more often switch to a tricyclic antidepressant. At UMCU, MAO inhibiters form a significant fraction of follow-up medication, including patients that tried an nSSRI, SSRI or tricyclic antidepressant first.

### Mental health recovery outcome measures

Examples of (translated) found sentences for each of the analyzed themes, core complaints, social functioning, well-being and patient’s experience, are “Anxiety complaints less than Tuesday last week and manageable”, “Patient says they have the feeling they are improving every day”, “Patient likes working more, because it improves daily structure” and “Patient experiences more peaceful feelings”. At PG, on average 4.06, 1.65, 4.04 and 5.69 sentences indicating a moment of change with respect to complaints, social functioning, well-being and experience were detected during antidepressant prescription periods. At UMCU, 11.2, 4.63, 10.0 and 16.7 sentences were on average detected for the respective themes. A possible explanation for this difference could be the nature of the patient reports at both centers: at the UMCU, follow-up was entirely inpatient, reflected in a higher mean number of days with available clinical notes (34) and total length of all clinical notes combined (mean 82.691 characters per patient). At PG the follow-up was mostly outpatient, where clinical notes were on average available on 28 days with a mean total length of 26.674 characters per patient.

### Bayesian networks

**Figure 3:**
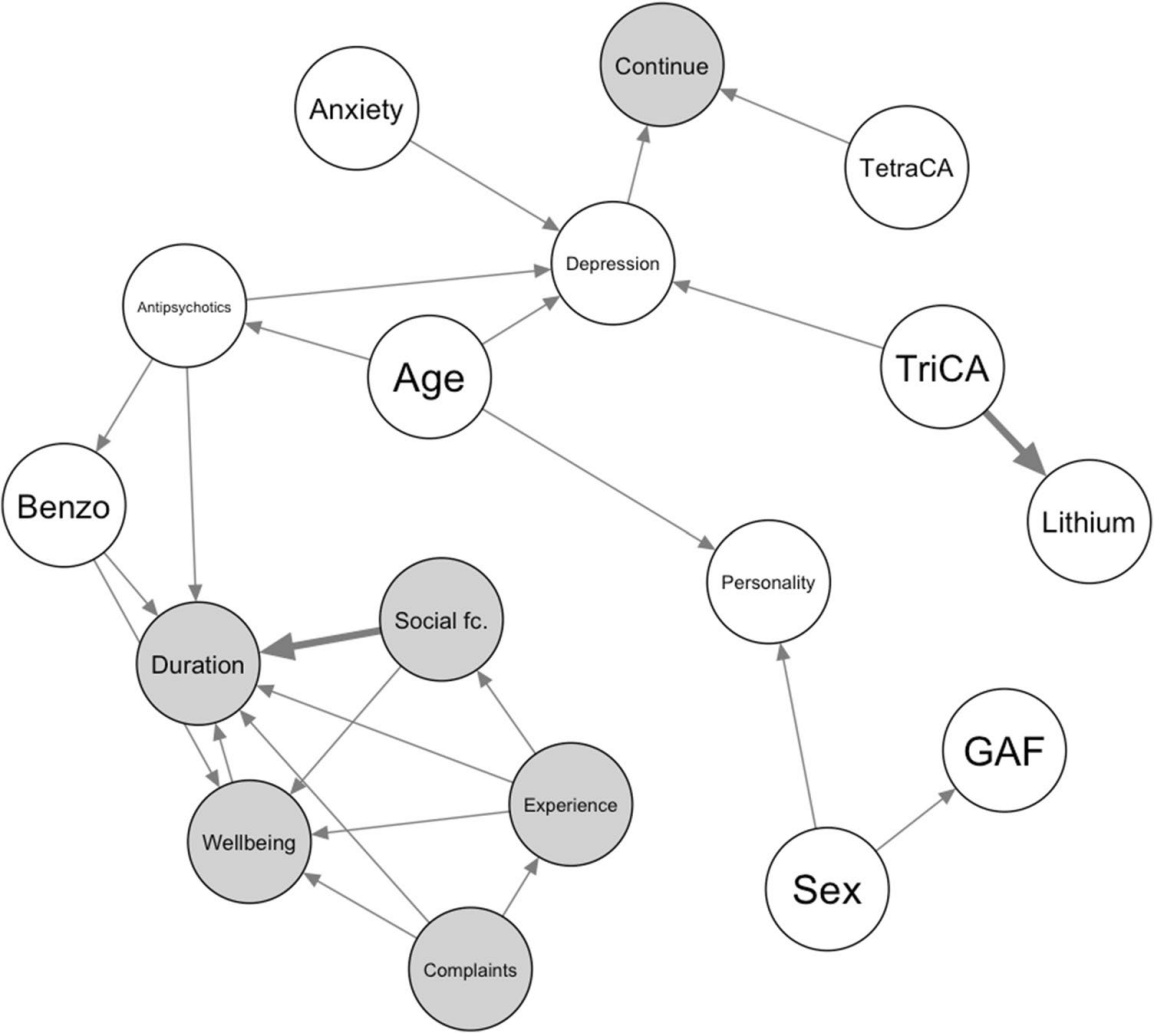
Bayesian networks found through constraint-based estimation (with the pcStable algorithm) on the PG data. Outcome variables are highlighted in grey. Dependencies that were also present in the UMCU network are highlighted with bold arrows. Abbreviations: Personality; personality disorder; Social fc: social functioning; Social pr.: social problems according to DSM.

With the constraint-based algorithm, the bootstrapped averaged network contained 24 arcs connecting the variables in the network for the PG data (Fig. 3). The UMCU bootstrapped averaged network only contained 9 arcs, of which 2 were also present in the PG network. Interestingly, many dependencies were found between the text-mined outcome measures and prescription duration nodes, indicating that trajectory outcomes are tightly intertwined. In the PG data, the use of benzodiazepines and antipsychotics during antidepressant prescriptions were directly linked to these outcomes. Whether a patient switched antidepressant types was directly dependent on TetraCA prescriptions and a DSM diagnosis of a (type of) depressive disorder.

**Figure 4:**
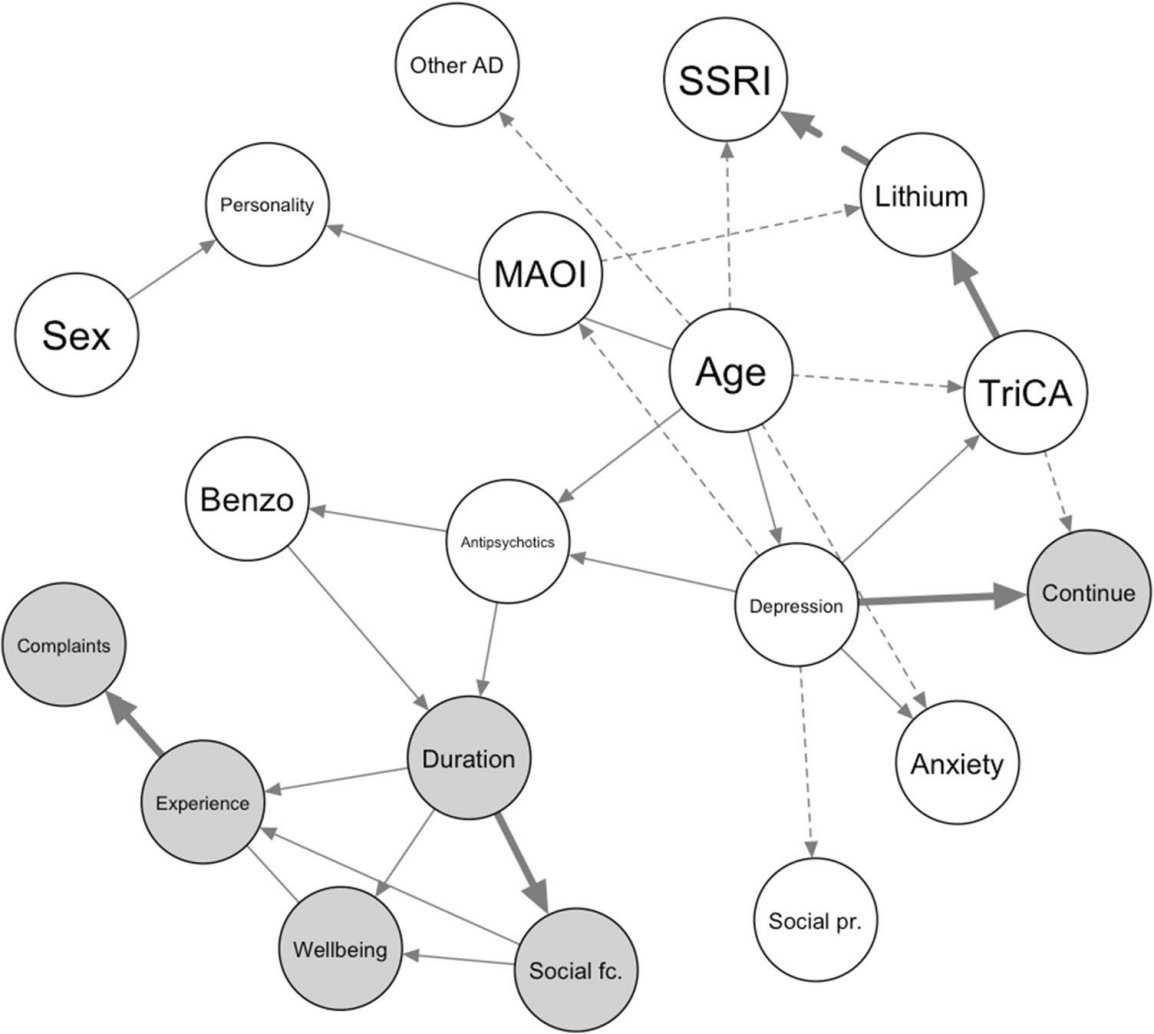
Bayesian networks found score-based estimation (with the tabu algorithm) on the PG data. Outcome variables are highlighted in grey. Dependencies that were also found for the UMCU data are highlighted in bold. Connections that were not found with the constraint-based method on the PG data are indicated with dashed lines. Abbreviations: Personality; personality disorder; Social fc: social functioning; Social pr.: social problems according to DSM.

The analysis was repeated with a score-based algorithm (tabu). In the PG data, 28 dependencies were found in the bootstrapped averaged network (see Fig. 4), of which 19 overlapped with the constraint-based network (irrespective of the direction of the dependency). In the UMCU data, only 9 dependencies were found, of which 5 overlapped with the PG network. The direct dependencies between benzodiazepines, antipsychotic prescriptions and the outcome measures remained present. A direct effect of tricyclic antidepressants on the probability of switching to another type of antidepressant was found in this network, instead of an indirect effect through the DSM diagnosis of a specific depressive disorder, and no dependency on the prescription of tetracyclic antidepressants was found.

### Found dependencies and comparison to expert opinion

Below a few examples are given of hypothetical patients and quantification of the dependencies found in the network. These patterns could give some interesting pointers for future confirmatory research. Note that even though sometimes variables are not direct neighbors in the network, they can depend on each other indirectly, in the absence of information on other nodes. For example, if we are unsure whether we are going to diagnose a patient as having depressive disorder as a main diagnosis, the patient’s age and DSM classification for social problems can give us extra information about whether the patient will switch antidepressants according to the score-based network. When we decide on the patient’s depression diagnosis, these paths become “blocked” and these variables do not give us extra information on a possible future switch anymore^29^.

Building on this example, we see that the network predicts that for patients with social problems that are also prescribed antipsychotics, the probability of choosing the correct antidepressant type (not having the switch) increases with 6% when they are prescribed a TriCA instead of another antidepressant type. This could potentially be explained by the fact that the prescription of anti-psychotics suggests severe, possibly psychotic, depression and TriCAs are more effective in more severe depression states, possibly due to anticholinergic effects that more strongly reduce stress or anxiety. For patients without social problems and without antipsychotics prescriptions, this “profit” after choosing a TriCA is even bigger and increases to 11%. A possible rationale could be the beneficial effect of TriCAs being explained by its anticholinergic, sedating properties, which would have a smaller effect on patients already taking antipsychotics^30^.

Focusing on the other outcome measures, we see that these are completely determined by each other and the decision to prescribe benzodiazepines and antipsychotics. If our hypothetical patient is treated with both benzodiazepines and antipsychotics, the probability of sufficient prescription duration to experience a clinical effect would be 76%. The probability of well-being having a positive sentiment score in clinical notes would be 52%. However, if our hypothetical patient is not going to take benzodiazepines and antipsychotics, the probability of continuing the initial antidepressant prescription beyond 5 weeks drops to 52%, and the probability of positive well-being scores drops to 46%. There appears to exist some interaction between benzodiazepine and antipsychotic use that strengthens or dominates the effect of the antidepressant prescribed which makes switching less necessary.

The outcome measures prescription duration, core complaints, social functioning, well-being and patient experience were tightly connected in the found networks. For example, a net positive well-being score improved the probability of obtaining a positive score on the core complaints domain with 15.2%. Incorporating the different domains in the network also allows for possible personalized recommendations in future decision support tools. For example, in the score-based network, adding benzodiazepine and antipsychotics to a treatment regimen only improved the probability of a positive net mean score on the complaints domain with 1.4%, but the social domain score improved with 6.9%. A patient that is especially interested in improving social functioning might find information on these outcome domains presented separately in a decision support tool especially useful, in contrast to a single pooled outcome measure.

## Discussion

This work in this manuscript concerned using Bayesian network analysis combined with NLP for pattern discovery in patient characteristics, treatment choices and outcomes during antidepressant trajectories. Several interesting trends were observed in the routinely collected clinical data studied. In the secondary and tertiary care settings studied, antidepressant choices had a higher continuation rate (66% and 89%) than expected from literature. Bayesian networks based on the data from PG, the secondary care mental healthcare facility, revealed 28 (predictive) dependencies between treatment choices, patient characteristics and outcomes. At UMCU, the tertiary care mental healthcare facility, most of these dependencies could not be replicated.

We have shown that using NLP to preprocess routinely collected clinical data can allow pattern discovery through Bayesian network analysis in a relatively big corpus of patient data. The combination with NLP enables large-scale studies without burdening clinical staff with extra administrative tasks for research purposes, such as separately registering patient prescriptions and specific treatment outcome measures. These tools could be combined in future research for investigating similar exploratory research questions. The possibility of using Bayesian network analysis for confirmatory research is discussed further below.

In patients who switched their antidepressant type during follow-up, switches were quite evenly distributed over other antidepressant types, although switching occurred more often after prescribing tetracyclic antidepressants or “other” antidepressants (for example bupropion) at PG. Interestingly, these types of antidepressants, frequently prescribed as third-line therapy options, appear slightly less effective in actual clinical practice. This leads to the hypothesis that specific subtypes of depression, perhaps not studied in clinical trials, must have different antidepressant working mechanisms. This makes it even more relevant to search for patterns that can predict the right prescription in an early phase of treatment.

Unfortunately, follow-up at the tertiary care facility UMCU was limited, possibly explaining the absence of most dependencies found at PG. As the network we are estimating here appears to be sparse and we do not expect variables in the network to have more than five predictive factors, the 735 patient trajectories used for learning at the tertiary care facility should have sufficed^18^, thus not explaining the missing dependencies. Another possible explanation could be the specialized nature of the care given at UMCU, with the different types of patients really having another network graph underlying the antidepressant trajectory data, where perhaps different variables should be included.

Patterns discovered in this study should be purely interpreted in an exploratory manner, as Bayesian networks require several assumptions to be met to enable causal interpretations^18^. Two important assumptions are that there should be no selection bias in the data, and there should be no (hidden) confounding variables. These are two assumptions that are very difficult to verify when working with retrospective data. Exploratory analysis did not show a confounding effect of treatment location on model outcomes (data not shown), but to truly fulfill these assumptions a randomized controlled trial should be performed where patients are randomly allocated to a (combination of) antidepressant(s). Such a trial would probably be unattainable in clinical practice because of ethical constraints (assigning a MAO inhibitor with severe potential side effects to someone with mild symptoms of depression, for example).

Nevertheless, unlocking additional data sources could already potentially improve the quality and value of exploratory analyses with the goal of pattern discovery such as the current study. We studied first-time inpatient antidepressant treatment trajectories, but limited information on the trajectory leading up to and following this inpatient treatment period, for example from general practitioners, was available in the data collected during inpatient care. It could possibly add a lot of value to incorporate antidepressant types tried before and after inpatient treatment into the model. Linking electronic patient files between general practitioners and mental health care facilities would enable this, but is currently not standard in the Netherlands. Future studies should also consider non-pharmaceutical therapies such as psychotherapy. Further, as selecting an antidepressant, especially in the inpatient setting, is a process where lots of factors, such as specific symptoms, side effects, earlier use of antidepressants, and patient’s preferences can be considered (at least according to Dutch guidelines)^31^, individual psychiatrists could potentially influence the choice and thus outcomes of antidepressant treatment^32^. Therefore, studies on the interplay between the specific psychiatrist prescribing the antidepressant, treatment choices made and treatment outcomes are warranted.

To formalize (confirm) the patterns found in the larger samples, a prospective study could eventually be conducted. This would enable precisely registering which antidepressants were actually administered, in addition to knowing which were prescribed. Further, this would allow a comparison with standard measuring instruments of depressive disorder. Such a prospective study could also enable the opportunity to include more detailed information on different symptoms patients are experiencing, and possible side effects of antidepressants. Since side effects are an important cause of discontinuation of antidepressants, the incorporation of different types of side effects could be of great value in the decision making about the type of antidepressant and medication adherence. Cipriani et al.^33^ found significant differences between types of antidepressants and continuation rates and highlighted the importance of strategies to distinguish differences in response to antidepressants on an individual level.

When it is not possible to collect this more detailed information on symptoms or side effects, possibly because of the high administrative burden of a prospective study with large sample sizes, feature extraction through natural language processing could potentially offer a solution. The NLP model used in this study was specifically developed to model broadly defined treatments for diverse groups of patients. More symptom-specific NLP models such as MedCAT could be used to extract specific symptoms or side-effects from routinely collected clinical notes^34^. A recent pilot study on using MedCAT for extracting information on cognitive side effects during depression treatment with electroconvulsive therapy showed promising results^35^. These approaches with reuse of clinical data could be of great value for personalized medicine because it will enable learning for a wider spectrum of patient types. For personalized modelling this is needed because the current strict selection criteria for patients to be included in clinical trials limits the extrapolation of study outcomes to individuals in daily practice^5^.

In the future, networks like the one described in this study could be translated to decision support tools in clinical practice. Individual patients could for example choose the outcomes they are most interested in, and the characteristics that influence the predicted outcomes for individual patients the most could be highlighted^36^. A systematic review of Samalin et al.^37^ showed positive effects of shared decision making interventions on medication adherence and depression outcome. A personalized tool to facilitate this process would be of great value^37^. Essential for such a decision support tool is the incorporation of prospectively collected data, and the incorporation of uncertainty estimates. Recent advances in the field of statistics have revealed new possibilities to give these kind of estimates, for example confidence sequences for discrete (conditional) independence relations that are robust under sequential testing^38^. Incorporating these into a Bayesian-network based clinical decision tool that is prospectively updated would enable offering patients and clinicians robust and up-to-date estimates. Hopefully, the techniques for finding new patterns in routinely collected clinical data presented in this work will ultimately contribute to the development of such new support tools.

## Supplementary Information

Supplementary Information.

## Data Availability

The data that support the findings of this study are available from UMCU and PG but restrictions apply to the availability of these data, which were used under license for the current study, and so are not publicly available. Data are however available from the authors (contact RT, r.j.turner@umcutrecht.nl or KH, k.hagoort@umcutrecht.nl) upon reasonable request and with permission of PG. Note: because of the nature of the data transfer agreement involved and the privacy regulations that are currently in place, it is not possible to transfer the data, but for example for replication purposes a guest appointment at UMCU and PG to analyze the data locally could be requested upon reasonable request.
