# Supplementary material for: Bayesian network analysis of antidepressant treatment trajectories
Source: Sci Rep. 2023 May 24;13:8428. doi: 10.1038/s41598-023-35508-7 (PMC10209137; doi:10.1038/s41598-023-35508-7)
Supplement: Supplementary file 1 — Supplementary Information. [file 41598_2023_35508_MOESM1_ESM.pdf]

Supplementary Material for Bayesian Network Analysis of Antidepressant Treatment Trajectories

Rosanne J. Turner*[1,2], Karin Hagoort[1], Rosa J. Meijer[3], Femke Coenen[1], Floortje E. Scheepers[1]

1. Department of Psychiatry, UMC Utrecht Brain Center, University Medical Center Utrecht, Utrecht University, 3584 CX Utrecht, The Netherlands
2. Machine Learning Group, CWI (national research institute for mathematics and computer science), Amsterdam, The Netherlands
3. Data Science Department, Parnassia Groep, Den Haag, The Netherlands

*corresponding author, r.j.turner@umcutrecht.nl

Supplementary table 1 Overview of antidepressant prescription groups and specific antidepressants present in the data

MAOI	Tranylcypromine
	Moclobemide
	Phenelzine
nSSRI	Trazodone
	Duloxetine
	Venlafaxine
Overig	Bupropion
	Vortioxetine
	Agomelatine
	Hyperici herba
SSRI	Sertraline
	Citalopram
	Escitalopram
	Fluoxetine
	Paroxetine
	Fluvoxamine
TetraCA	Mirtazapine
	Mianserine
TriCA	Nortriptyline
	Amitriptyline
	Clomipramine
	Imipramine
	Doxepine
	Maprotiline
	Dosulepine

Supplementary table 2 Overview of therapeutic dose range for selection of antidepressant treatment trajectories

antidepressant	**Minimal dose**	**Maximal dose**
tranylcypromine	10	60
phenelzine	8	120
moclobemide	100	600
clomipramine	10	250
nortriptyline	20	250
amitriptyline	10	150
imipramine	10	300
dosulepin	50	225
doxepin	25	300
trimipramine	NA	NA
venlafaxine	75	375
mirtazapine	15	45
trazodone	100	400
bupropion	150	300
duloxetine	60	120
agomelatine	25	50
vortioxetine	5	20
hyperici herba	NA	NA
sertraline	50	200
citalopram	10	40
fluoxetine	20	60
escitalopram	5	20
paroxetine	20	50
fluvoxamine	50	300

Supplementary table 3 Detailed summary of outcome measures per antidepressant prescription group.

AD type	*Facility*	*N*	*Continuation*	*If switched: median duration until switch (days)*	*Prescription duration*	*Core complaints*	*Social*	*Well-being*	*Experience*
SSRI	PG	2244	0.680	77	162	-0.166	0.337	0.301	-0.084
	UMCU	316	0.924	16	92	-0.344	0.386	0.094	-0.217
nSSRI	PG	774	0.625	97	188	-0.174	0.324	0.302	-0.117
	UMCU	147	0.878	21	143	-0.119	0.567	0.229	-0.1128
TriCA	PG	853	0.742	86	175	-0.117	0.322	0.257	-0.077
	UMCU	192	0.901	42	122	-0.098	0.493	0.201	-0.079
TetraCA	PG	827	0.573	45	126	-0.115	0.280	0.308	-0.115
	UMCU	44	0.886	51	49	-0.182	0.689	0.140	-0.222
MAOI	PG	62	0.613	122	212	-0.102	0.167	0.250	0.101
	UMCU	45	0.733	14.5	137	-0.057	0.390	0.187	-0.121
Other	PG	224	0.558	85	170	-0.180	0.399	0.263	-0.147
	UMCU	15	0.733	14.5	54	-0.340	0.432	0.105	-0.317

Note that 171 out of 4808 trajectories at PG and 24 at UMCU concerned trajectories where two types of antidepressants were started on the same day. At PG, 106 concerned combinations of a tetracyclic antidepressant with another type; at UMCU this concerned 12 of the 24 cases. The remainder mainly consisted

of combined prescriptions of tricyclic antidepressants, SSRIs and nSSRIs, possibly discontinuation schemes started at the beginning of the admission of the patient. For the outcome measure summaries in this table, if a patient started two types of antidepressants at the same day, this data is incorporated in the two separate corresponding rows in the table. This separation into two entries is offered here purely with the purpose of keeping this table concise. In the Bayesian network analyses in this manuscript, these types of trajectories are viewed as one trajectory with a combination of antidepressant types: the Bayesian network can handle learning such interactions between variables in the model.
